# The Evolution of Cystic Echinococcosis in Humans and Ruminants in Portugal—A One Health Approach

**DOI:** 10.3390/vetsci10090584

**Published:** 2023-09-21

**Authors:** Ana Margarida Alho, Miguel Canhão Dias, Miguel Cardo, Pedro Aguiar, Luís Madeira de Carvalho

**Affiliations:** 1 Northern Lisbon Public Health Unit Francisco George, 1549-010 Lisbon, Portugal; margarida.alho@arslvt.min-saude.pt; 2 NOVA National School of Public Health, Public Health Research Center, Comprehensive Health Research Center, CHRC, NOVA University Lisbon, 1600-560 Lisbon, Portugal; pedroaguiar@ensp.unl.pt; 3 Global Health and Tropical Medicine, GHTM, Instituto de Higiene e Medicina Tropical, IHMT, Universidade NOVA de Lisboa, 1349-008 Lisbon, Portugal; 4 CIBIO, Centro de Investigação em Biodiversidade e Recursos Genéticos, InBIO Laboratório Associado, Campus de Vairão, Universidade do Porto, 4485-661 Vairão, Portugal; 5 Departamento de Biologia, Faculdade de Ciências, Universidade do Porto, 4099-002 Porto, Portugal; 6 BIOPOLIS Program in Genomics, Biodiversity and Land Planning, Centro de Investigação em Biodiversidade e Recursos Genéticos CIBIO, Campus de Vairão, 4485-661 Vairão, Portugal; 7 CIISA—Centre for Interdisciplinary Research in Animal Health, Faculty of Veterinary Medicine, University of Lisbon, 1300-477 Lisbon, Portugal; mjcardo@fmv.ulisboa.pt; 8 Associate Laboratory for Animal and Veterinary Sciences (AL4AnimalS), 1300-477 Lisbon, Portugal

**Keywords:** *Echinococcus granulosus*, hydatid cyst, hospitalizations, slaughtered animals, public health, zoonosis, prevalence

## Abstract

**Simple Summary:**

Cystic echinococcosis, also known as hydatid disease, is a significant parasitic infection that affects animals and humans. In Portugal, there is limited information available about this disease despite its importance to public health. To better understand its impact, we collected data from ruminant slaughterhouses, human hospitalizations, and human-confirmed infection cases. Between 2008 and 2022, 298 cases of cystic echinococcosis were identified in ruminants slaughtered for human consumption from national farms in Portugal. Of these, 192 cases were in sheep, 95 in cattle, and 11 in goats. Among humans, the disease resulted in 582 hospitalizations, with an average stay of 11 days, and caused 13 deaths between 2008 and 2018. We found that each infected animal increased the likelihood of human hospitalization by 7%. Additionally, for every 100,000 person-years observed during the same period, there were approximately 0.528 hospitalizations. Living in the Alentejo region was associated with a 5.3-fold increase in hospitalization rates and an 8-fold higher risk of death from cystic echinococcosis. This collaborative One Health study provides valuable insights into the situation in Portugal and highlights the need for effective health control programs to prevent the spread of this neglected zoonosis.

**Abstract:**

Cystic echinococcosis, also known as hydatid disease, is a significant parasitic zoonosis with public health implications, albeit often neglected. In Portugal, data on this zoonosis are scarce despite being a mandatory notifiable disease in both humans and animals. To assess the impact of cystic echinococcosis on both livestock and humans, we compiled data from slaughterhouse records of ruminants, human hospitalizations, and confirmed cases of human echinococcosis. Overall, a total of 298 cases of cystic echinococcosis were identified in ruminants slaughtered from national farms for human consumption in Portugal between 2008 and 2022, comprising 192 cases in ovines, 95 in bovines, and 11 in caprines. Echinococcosis led to 582 hospitalizations in Portuguese public hospitals, with an average hospital stay of 11 days (±15.66), and resulted in 13 deaths (2.23%) from 2008 to 2018. Each infected animal was associated with a 7% increase in the incidence rate of human hospitalization (*p* = 0.002, IRR = 1.070, 95% CI: 1.025–1.117). Additionally, for every 100,000 person-years observed between 2008 and 2018, the total number of hospitalizations was 0.528. Residence in the Alentejo region was associated with a 5.3-fold increase in the incidence rate of human hospitalizations and an 8-fold higher risk of death from echinococcosis.

## 1. Introduction

Echinococcosis is an important parasitic zoonosis, considered by the World Health Organization (WHO) one of the 20 neglected tropical diseases with significant global health implications [1]. Cystic echinococcosis or hydatid disease is caused by the taeniid cestode *Echinococcus granulosus* sensu lato. Hydatid cysts, which are the larval stage of *Echinococcus granulosus* s.l., develop in intermediate hosts and humans when they ingest eggs that have been excreted by canids in their feces. The severity of the disease depends on the number, size, and location of the cysts. Humans are considered dead-end hosts for cystic echinococcosis, with the transmission of the infectious parasite eggs mostly by hand-to-mouth or by food or water. In humans, hydatidosis can be asymptomatic but also fatal [2]. Apart from a public health and animal health concern, hydatidosis in domestic ruminants also causes significant economic impact due to the condemnation of damaged organs and decreased production of meat, milk, and wool [3].

Overall, echinococcosis has a worldwide distribution, with Mediterranean countries known as one of the most affected in the European Union [4]. The *Echinococcus* genus is present in every continent (except for Antarctica) and has a significant impact in some of the poorest areas of the world, namely East Africa and Central Asia. In many countries, however, there is still a lack of precise notification of the disease, hampering efforts to map the disease’s distribution [4]. In the European Union, the notification rate of the disease in humans has been dropping and currently sits at 0.15 cases per 100.000 inhabitants [5].

In Portugal, only *Echinococcus granulosus* has been recorded, with 646 cases registered in humans between 1979 and 2008 [6] and 91 between 2002 and 2016 [7,8,9,10]. Although the Alentejo region has historically been identified as a hyper-endemic area of the disease, it has been suggested that high incidences are mainly localized in the district of Évora [6]. While there are 10 described strains (G1–G10), each having differing pathogenicity levels and preferential hosts, only G1, G3, and G7 have been identified in Portugal in humans, cattle, sheep, goats, and wolves [11,12,13,14].

Despite human and animal echinococcosis being closely linked, epidemiological data from the human and veterinary fields are still segregated, jeopardizing the effectiveness of control measures and the identification of risk factors and areas where interventions may be required. Additionally, data in Portugal are still scarce and punctual. Thereby, a retrospective nationwide study was conducted by a multidisciplinary team, bringing together the expertise of public health doctors, veterinarians, and epidemiologists to assess the impact of this zoonosis on humans and livestock.

## 2. Materials and Methods

### 2.1. Animal Data

Slaughterhouse records from domestic livestock (bovine, caprine, and ovine species) were obtained from the official Portuguese Directorate-General for Food and Veterinary Affairs (Direção-Geral de Alimentação e Veterinária, DGAV, Lisboa, Portugal). Data refers to all the slaughters that occurred in Portugal’s mainland between 2008 and 2022. Livestock meat inspection was carried out by official veterinarians who are in charge of *ante-mortem* and *post-mortem* inspection, food safety procedures, and animal welfare. These professionals are trained to identify potential lesions in tissues that are unfit for human consumption, or that could pose a risk to public health, ensuring their removal from the food chain [15].

Meat examination consists of visual, palpatory, and incision inspection techniques, as imposed by the European Union’s legal requirements. For the presence of parasitic lesions compatible with echinococcosis, the liver and lungs are routinely examined. Whenever a suspected lesion is found, it is sent to the National Veterinarian Reference Laboratory for histopathological examination of the detected lesions. All inspection findings are reported in an electronic platform (Information System for the Approval and Control Plan for Establishments—Sistema de Informação do Plano de Aprovação e Controlo dos Estabelecimentos, SIPACE) that stores all nationwide condemnation data.

To ensure an accurate identification of the origin of the slaughtered animals in different geographic areas, a detailed survey was conducted to determine the district and municipality of origin for each farm. Since animals may pass through multiple farms before arriving in the slaughterhouse, an analysis was performed to trace the path of each affected animal. For bovines, the farm where the animal resided the longest was recorded as its place of origin. In contrast, for ovines and caprines, tracing the origin of all animals with hydatid lesions was not feasible. In such cases, the farm that presented the affected animal for slaughter was considered its place of origin.

### 2.2. Human Data

Anonymized records of officially confirmed human cases of echinococcosis were obtained from the Portuguese Directorate-General of Health (Direção-Geral da Saúde, DGS, Portugal) for the period from 2009 to 2018. These cases were reported through the National Epidemiological Surveillance System (Sistema Nacional de Vigilância Epidemiológica, SINAVE). To qualify as a human case of echinococcosis, at least one of the following criteria must be met: (a) Histopathology or parasitology that aligns with *E. granulosus* (for instance, the direct observation of the proto-scolex in cystic fluid); (b) Identification of *E. granulosus* cysts featuring pathognomonic macroscopic morphology in surgical specimens; (c) The presence of typical organic lesions revealed through imaging techniques (e.g., computed tomography, ultrasound, magnetic resonance imaging) along with serological test confirmation; (d) Detection of *Echinococcus* spp. specific serum antibodies via a highly sensitive serological assay (ELISA, ELIEDA, HA, IF, etc.) confirmed by a highly specific serological test (Immunoblot); or Detection of nucleic acids from *E. granulosus* in a biological sample (Order No. 1150/2021).

Additionally, anonymized human hospitalization records from Portuguese National Health Service hospitals were obtained from the Health System’s Central Administration (Administração Central do Sistema de Saúde I.P., ACSS, Lisboa, Portugal). Hospitalizations having cystic echinococcosis as the main cause of admission, based on the International Classification of Diseases ICD-9/ICD-10, were assessed.

### 2.3. Statistical Analysis

A descriptive characterization of meat inspections was conducted by animal species, year, municipality, and NUTS II region of the farm’s location. Similarly, a descriptive characterization of human cases was also performed by year, sex, age group, contact with animals, district, and municipality of residence. For human hospitalizations, a descriptive analysis was conducted by year, composed of the absolute and relative frequencies stratified by sex, age group, days of hospitalization, and region, district, and municipality of residency. In order to account for the effect of population size on the number of hospitalizations, we used data from Census 2021, provided by the Portuguese National Institute of Statistics (Instituto Nacional de Estatística, INE, Portugal) [16], dividing the number of hospitalizations in each region by its population and multiplying by 1,000,000, allowing a more contextualized analysis of absolute frequencies per million inhabitants. All analyses were conducted using SPSS^®^21 for Windows.

To investigate the association between the outcome variable and relevant covariates, and as we were dealing with rare events, a Poisson log-linear regression model was used with a logarithmic link function [16]. Two predictors were considered: “the total number of infected animals” and “living in the region of Alentejo”. The offset variable was the logarithm of the population resident in each district in each year from 2008 to 2018. A robust estimator was adopted to adjust for eventual assumption issues. With this model, we achieved incidence rate ratios of hospitalized people, according to each of the predictors, with a significance level of 0.05. For this Poisson log-linear regression model, data from the period between 2008 and 2018 were analyzed using two databases: slaughterhouse animal records and human hospitalizations. The number of officially confirmed human cases was so low that it was not possible to detect any relevant level of association. Information on the demographic characteristics of the human population was obtained from the annual intercensus estimations and decennial censuses from INE [17].

### 2.4. Spatial Analysis

A Geographic Information System-based modeling was used to map the origin of animal cases and human hospitalizations. Only data from farms in Portugal, as well as hospitalizations and cases in Portugal, were mapped. We utilized an online mapping tool to visualize the distribution of cases, employing the website Map in Seconds [18].

## 3. Results

### 3.1. Cases of Animal Echinococcosis—Slaughterhouses Records

From 2008 to 2022, out of the 17,180,241 ruminants slaughtered in Portugal, 427 cases of echinococcosis were found. Of this total, 298 cases were found in animals bred on Portuguese farms, and 129 cases were found in animals that came from Spanish farms.

Of the 298 cases from Portuguese farms, 192 occurred in ovines, 95 in bovines, and 11 in caprines (Table 1). Of the 129 cases from Spain, 96 were documented in ovines, 30 in bovines, and 3 in caprines.

**Figure 1:**
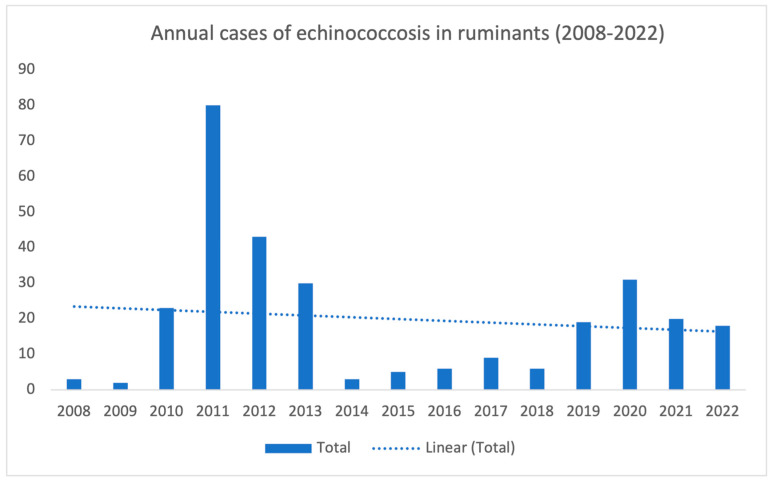
Annual number of cases of cystic echinococcosis detected in ruminants raised on Portuguese farms through *post-mortem* inspections between 2008 and 2022. Total of positive ruminants—298.

When analyzing national slaughter data over the years, there is no evident decrease in the overall number of animals with cystic echinococcosis lesions detected during post-mortem inspection. However, it is possible to observe a downward trend, as illustrated in Figure 1.

**Figure 2:**
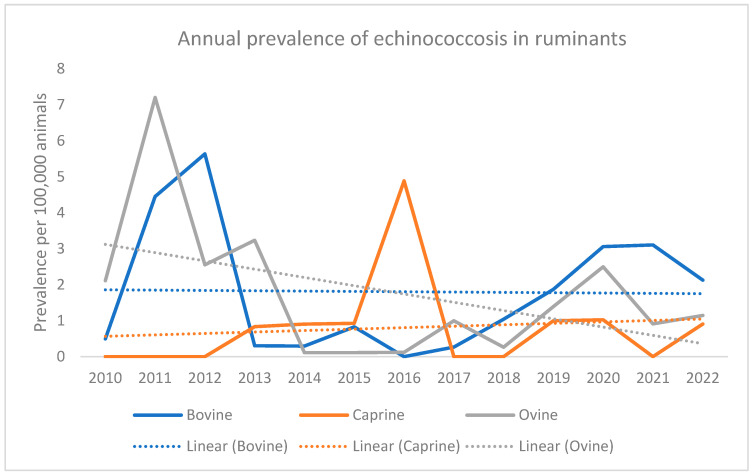
Annual prevalence per 100,000 animals of cystic echinococcosis through *post-mortem* inspections in bovines, ovines, and caprines in Portuguese slaughterhouses between 2008 and 2022.

Despite occasional fluctuations across the years, the overall prevalence of echinococcosis in all three species remained low, ranging from 0 to a maximum of 7.2 cases per 100,000 animals. Longitudinal analysis indicates a decreasing trend in ovines, a slight increase in caprines, and a stabilizing trend in bovines, as illustrated in Figure 2. The overall prevalence of echinococcosis was found to be 1.83 cases per 100,000 bovines, 1.78 cases per 100,000 ovines, and a notably lower prevalence of 0.76 cases per 100,000 caprines.

Out of the 298 cases from national farms and considering the five mainland Portuguese administrative NUTS II regions (North, Centre, Lisbon Metropolitan Area, Alentejo, and Algarve), Alentejo accounted for the highest number of detected cases, with a total of 161 positive animals (54%). The Centre region reported 80 cases (26.8%), followed by 39 cases in the North region, 17 cases in the Algarve region (5.7%), and 1 case in the Lisbon Metropolitan Area.

**Figure 3:**
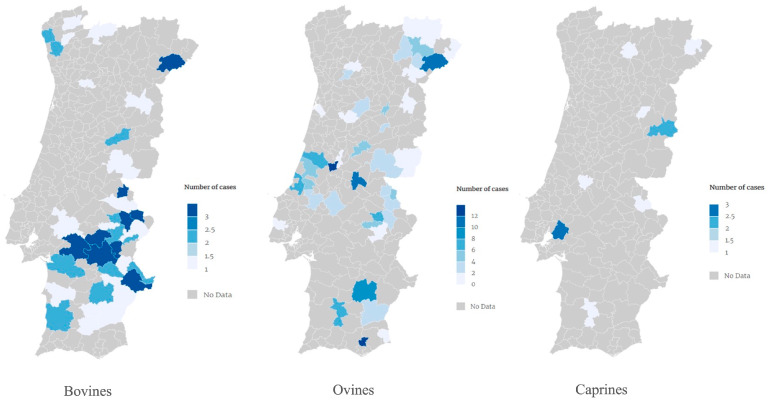
Geographical distribution of the 298 cases of cystic echinococcosis detected in ruminants raised on Portuguese farms through *post-mortem* inspections between 2008 and 2022 by municipality.

More specifically, spatial analysis confirmed that the Portalegre district had the highest number of ruminant cases across all animal species (n = 72), followed by Évora (n = 44), and then by the districts of Leiria, Beja, and Bragança with 39, 33, and 28 cases, respectively (Figure 3 and Supplementary Table S1).

### 3.2. Cases of Human Echinococcosis

**Figure 4:**
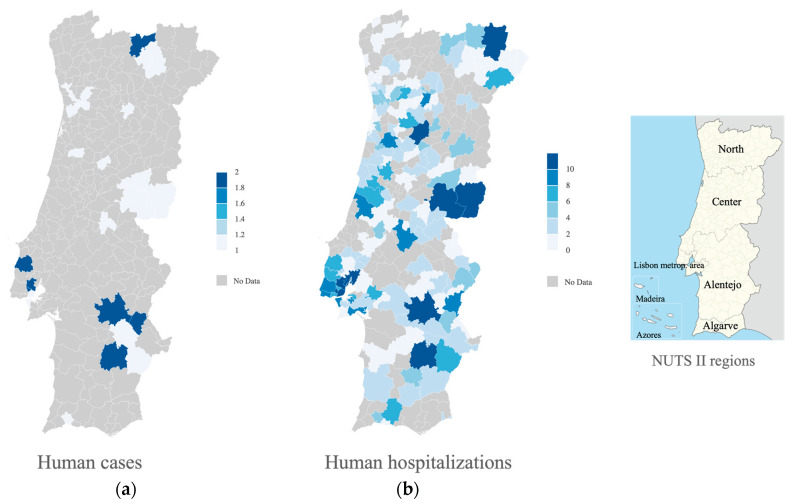
(**a**) Map of human cases of echinococcosis by patients’ municipalities of residence; (**b**) Map of human hospitalizations due to echinococcosis by patients’ municipalities of residence (2008–2018). NUTS II region boundaries.

A total of 35 cases of human echinococcosis were reported in Portugal and validated by the national authority from 2009 to 2018. Of these, 16 occurred in males and 19 in females. Concerning age, 11.43% of the cases occurred from 10–19 years old, 5.71% from 20–29, 8.57% from 30–39, 20% from 40–49, 20% from 50–59, 14.29% from 60–69, and 20% in individuals from 70–79 years old. Out of the 35 cases, the majority were hospitalized (62.86%, n = 22). Concerning risk factors, 37.14% referred to frequent contact with dogs, 8.57% potential egg ingestion through contaminated waters and 54.29% of the respondents said it was unknown. Most of the cases were from Lisbon district (20%), followed by Évora (17.14%) and Beja (11.43%) (Figure 4a).

### 3.3. Human Hospitalizations Due to Echinococcosis

**Figure 5:**
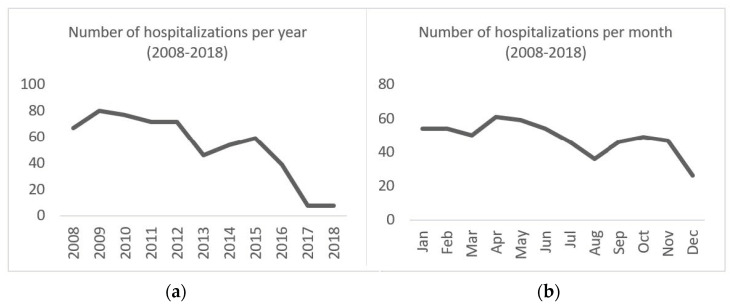
(**a**) Human hospitalizations due to echinococcosis per year (2008–2018); (**b**) Human hospitalizations due to echinococcosis per month (2008–2018).

Between 2008 and 2018, cystic echinococcosis was the main cause of admission in 582 hospitalizations (Figure 4b), 287 (49.3%) of which were male patients and 295 (50.7%) of female patients. The mean age of hospitalized patients was 56 (±17.58) years, with a minimum of 0 and a maximum of 93 years. The average length of hospitalization was 11 (±15.66) days, with a minimum of 0 and a maximum of 248 days. The total number of hospitalization days was 6601, and the disease was responsible for 13 deaths (2.23%). Overall, a decreasing trend in hospitalizations caused by echinococcosis was observed (Figure 5a). A heterogeneous monthly distribution of cases was found (Figure 5b).

**Figure 6:**
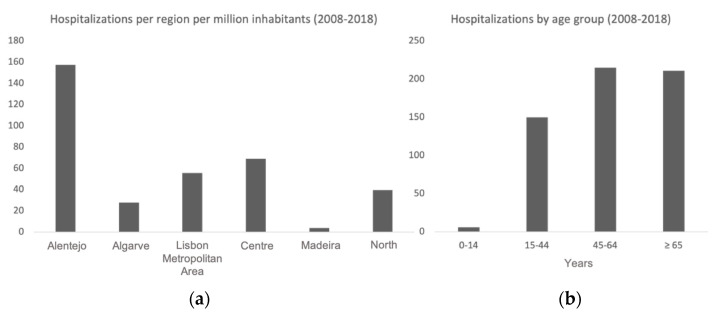
(**a**) Human echinococcosis hospitalizations in Portugal by NUTS II region, per million inhabitants (2008–2018); (**b**) Human echinococcosis hospitalizations by age group (2008–2018).

The Portuguese NUTS II region with the highest number of records of hospitalization per million inhabitants was Alentejo (Figure 6a). The age group with the highest number of hospitalizations was 45–64 years old (Figure 6b).

Based on our analysis of the number of infected animals, we found a significant 7% increase in the incidence rate of human hospitalizations. This means that for every infected animal, there was a 7% increase in the incidence rate of human hospitalization (*p* = 0.002, IRR = 1.070, 95% CI: 1.025–1.117). Furthermore, our study revealed that residing in the Alentejo region increased the incidence rate of human hospitalizations by 5.3 times when compared to other regions of continental Portugal (*p* < 0.001, IRR = 5.290, 95% CI: 3.835–7.296). This indicates that for every human hospitalization outside of the Alentejo region, there were more than five hospitalizations in the Alentejo.

Regarding animal species, there was a significant statistical association between the number of infected bovines and the number of human hospitalizations (*p* < 0.001, IRR = 1.313, 95% CI: 1.195–1.443). However, we did not find a significant association for either ovines (*p* = 0.210, IRR = 1.037, 95% CI: 0.980–1.099) or caprines (*p* = 0.451, IRR = 1.121, 95% CI: 0.833 –1.507). Interestingly, when we controlled for living in the Alentejo region, we found that the strength of association was much higher for the intrinsic characteristics of Alentejo (*p* < 0.001, IRR = 5.257, 95% CI: 3.715–7.438) than for the animal species (*p* = 0.927, IRR = 1.002, 95% CI: 0.956–1.050).

Furthermore, we estimated that for every 100,000 person-years observed between 2008 and 2018, there were a total of 0.528 hospitalizations. Additionally, the risk of death from echinococcosis was eight times higher for those living in the Alentejo compared to those living in other areas of continental Portugal (*p* < 0.001, IRR = 8.259, 95% CI: 2.427–28.106).

## 4. Discussion

Overall, out of the 17,180,241 ruminants slaughtered in Portugal between 2008 and 2022, 427 cases of echinococcosis were found. Of this total, 298 cases were found in animals bred on Portuguese farms, most of them in ovines (n = 192), followed by bovines (n = 95) and caprines (n = 11). The study revealed a generally low prevalence of echinococcosis across different types of livestock bred in Portugal, with bovines and ovines experiencing higher rates compared to caprines. This comprehensive analysis provided a thorough quantification of the prevalence and distribution of cystic echinococcosis in ruminants over an extended period. Although echinococcosis is a disease that must be reported in animals, prevalence studies at the slaughterhouse level are limited in most European countries. Recent studies conducted in other Mediterranean countries, such as Italy and Greece, have shown high prevalence levels in small ruminants, as well as in cattle in endemic/hyperendemic areas like Australia. These findings emphasize the need for more comprehensive studies [19] similar to the one conducted in Portugal.

This study also allowed the identification of risk areas for echinococcosis in Portugal, which is a crucial step in promoting public health strategies aimed at controlling this zoonotic infection. Notably, the Alentejo region, located in the south of Portugal, stood out as the location with the highest number of animal and human cases. This region has always been considered an endemic zone based on several epidemiological studies conducted since the 1970s. Alentejo is a rural area characterized by extensive free-range beef and sheep farms for meat production, in contrast to the other regions where farms are generally smaller, and animals are primarily kept indoors or in semi-intensive settings for reproduction or milk production. Besides, in Alentejo, there is also close contact between livestock and shepherd dogs, along with wildlife-livestock interactions that facilitate the transmission of pathogens [20]. Indeed, living in the Alentejo region has been shown to be a significant risk factor for human echinococcosis. Several factors contribute to this observation, including the common traditional practice of extensive farming in Alentejo, where ruminants are permitted to graze freely in natural pastures without effective movement control. This perpetuates a cycle of transmission: if a domestic or feral/wild canid becomes infected with *E. granulosus*, it will excrete eggs onto the pasture through feces, thereby contaminating the soil, water, and vegetation. This, in turn, leads to the infection of grazing animals. One possible contributing factor to this problem may lie in the lower frequency of deworming for cestodes in working, hunting, and shepherd dogs compared to pet dogs. The presence of stray dogs and feral canids in the area is another concerning factor. The roaming behavior of these dogs may contribute to the wider dispersion of zoonotic parasites, including their spread from hyperendemic to fewer endemic areas within the Alentejo region, as observed in our study and in other geographical zones [21,22,23]. Additionally, a common practice that may contribute to transmission is the provision of raw animal viscera to dogs by owners and shepherds. Although this practice has been discouraged through awareness campaigns conducted by veterinary services on infected farms, it may persist in certain areas. Other potential contributing factors include the presence of a predominantly elderly population, the rural environment, low literacy levels, and limited access to timely and regular healthcare. These factors may lead to delayed diagnosis in humans and a higher rate of hospitalizations [6]. As echinococcosis progresses untreated, the size of the cysts increases, and the parasites continue to multiply and grow inside the affected organs, leading to damage to the surrounding tissues and further complications. Despite improvements, certain geographical areas have remained hyperendemic, as evidenced by the persistence of human hydatid disease in the Évora district. In fact, 35.4% of suspected cases and 85.3% of monitored cases after surgery analyzed in reference laboratories originated from this district [6,14,24,25,26]. Therefore, our findings indicate that the issue persists in Alentejo, and effective control measures are not in place at the human level. The risk of infection, hospitalization, and mortality is significantly higher in this region compared to the other areas surveyed in our study.

Although the number of reported human cases and hospitalizations due to echinococcosis is relatively low, there may be a problem with underreporting. This may be caused by a high proportion of asymptomatic carriers, nonspecific symptoms, and a long incubation period in humans. It is also reasonable to assume that potential infections were not considered, by lack of diagnosis or reporting, given the subclinical and prolonged course of the disease, which may not present an acute onset and hence urgent hospitalization. According to Casulli et al., 2022 [27], 502 cases were detected in Portugal between 1997 and 2021, averaging 20 annual cases of cystic echinococcosis and a mean annual incidence of 0.19. While cystic echinococcosis remains endemic and neglected in several European regions, a general decrease in human incidences has been observed [27]. This trend has also been noted in Portugal. Various factors contribute to this decline, such as national epidemiological surveillance and control programs, educational campaigns, migration from rural to urban areas, improved hygiene practices, and intensified farming [28]. Nevertheless, echinococcosis remains one of the most neglected parasitic diseases, receiving little attention in terms of the development of new drugs and treatment approaches. Additionally, there are discrepancies between reported and actual cases, along with a lack of appropriate diagnostic tools in endemic areas. It is thereby crucial to raise awareness about the disease to ensure that patients receive appropriate treatment, as emphasized by human surgeons in Portugal [29,30].

Until recently, *E. granulosus* was the only species reported in our country, primarily associated with ruminant and human infections. However, recent findings have indicated the presence of the G7 genotype in wolves and cattle, introducing new aspects to the parasite’s presence in Portugal. These findings suggest the existence of two possible epidemiological cycles: one in the south, involving the interaction between dogs and ruminants, and another in the center-north, involving dogs/wolves and swine [11,12,13,24]. In 2021, hydatid cysts were reported for the first time in the liver of a free-living wild boar (*Sus scrofa*) in Portugal, and these metacestodes were identified as *Echinococcus ortleppi*. This marked the first report of this species in Portugal and Europe, highlighting the role of wildlife in the survival and spread of the *Echinococcus* genus [31]. Consequently, the biology and epidemiology of *Echinococcus* spp. in Portugal are continuously evolving, particularly those associated with wildlife. These factors can impact both animal and human infections and overall prevalence, emphasizing the need for Portuguese public health authorities to be vigilant and prepared for these new scenarios.

Some limitations of this study should be highlighted. Regarding meat inspection in slaughterhouses, not all identified echinococcosis lesions were confirmed through laboratory analysis. As a result, there is a possibility that some of the identified lesions may be caused by other conditions that resemble echinococcosis. Additionally, due to the reliance on visual, palpatory, and incision inspection techniques during meat inspection, it is also possible that some cases of echinococcosis may go undetected. Concerning human hospitalization records, despite being systematically collected and standardized, they depend on the medical records’ accuracy and coding and only reflect the reality of public hospitals, which account for 79% of all inpatient discharges, missing private healthcare institutions [17]. Besides, only data from Portugal’s mainland was available, leaving out the archipelagos of Madeira and the Azores. Moreover, data from the years 2017 and 2018 were not definitive.

Overall, our study revealed a decreasing trend in the number of cases of echinococcosis documented in ruminants over the years. This decline can be attributed to the implementation of legal procedures. Whenever a positive case of echinococcosis is detected in slaughterhouses, it is reported in the electronic platform and thoroughly investigated by the official animal health services. An evaluation is conducted, considering the movement of the affected animals, and local veterinary services carry out deworming campaigns for dogs in the areas where the animals have spent most of their productive lives. Locations, where animals spend most of their productive lives, are considered an important factor for vertebrate intermediate host infection with other cestode-associated zoonosis in Portugal, such as *Taenia saginata*/*Cysticercus bovis* in bovines. In this cestode, more than 6 weeks on the same farm would imply a 12-fold increase in prevalence compared with a period of less than 6 weeks [19,32,33],

In addition, various public health education measures have been promoted. These include collecting dog feces on farms, regularly analyzing fecal samples from at-risk dogs (such as shepherds, hunting dogs, and working dogs), deworming dogs every six weeks, and implementing appropriate biosecurity measures to prevent contact between livestock and wild carnivores. Other guidelines involve restricting home slaughter of sheep and other livestock, prohibiting the feeding of carrion or animal viscera to dogs, controlling stray dogs, and avoiding the consumption of food or water contaminated with dog feces. Regular handwashing with detergent and thoroughly cooking food are also advised. Given these preventive measures, the detection of animal cases of cystic echinococcosis in slaughterhouses should be of significant concern for public health [19,32].

Portuguese veterinary services also control echinococcosis by distributing antiparasitic pills (Praziquantel) to dogs during anti-rabies vaccination campaigns. In high-incidence areas, dogs receive a dose based on their weight, and a second dose is provided to the animal keeper for later administration. However, this dosage may be insufficient due to the short prepatent period of *E. granulosus,* and frequent treatments are necessary [32]. In urban settings, Portuguese dog owners have close physical contact with their pets, with 43.1% allowing dogs in their beds and 75.5% permitting licking of their faces, increasing the risk of zoonotic transmission [34]. In Lisbon, although 89.7% of owned dogs received anti-endoparasite treatment, only 11.8% followed the recommended quarterly regimen. This research was conducted after the economic crisis in 2008–2013 in Portugal, which may explain the low compliance among small animal owners towards effective parasite control programs with regular dewormings by that time. This lack of compliance might also account for the observed increase in cystic echinococcosis in ovine and bovine found in 2011–2013, namely in rural and low-income household zones such as in Alentejo [35]. A survey of municipal veterinary practitioners revealed a wide range of deworming frequencies and anthelmintic drugs used. Additionally, there was a high percentage of non-disposal of feces (>80%) and a low rate of post-deworming coprological analysis (around 20%) [36]. Finally, deficient parasite control may occur because of irregular/low frequency deworming, but also due to anthelmintic resistance. A recent study found resistance to Praziquantel in another dog cestode, *Dipylidium caninum.* Given that this drug has been in use for *E. granulosus* control since the 1970s, the possibility of resistance emerging must not be discarded in Portugal for cestodes [37].

These issues need to be addressed to effectively control dog echinococcosis and prevent cystic echinococcosis in humans and ruminants in endemic or hyperendemic areas. The geographic identification conducted in this study helps target awareness campaigns, such as regular deworming every six weeks for dogs in contact with livestock, as recommended by the European Scientific Council for Companion Animal Parasites in endemic areas [32]. But more attention should be placed on improving communication about this problem in elementary schools located in endemic areas. In fact, a study conducted in 2008 in the Alentejo region showed that almost 100% of the children were able to correctly recognize most of the preventive behaviors regarding this disease. Therefore, school training of young children remains a good strategy and may still contribute nowadays to better knowledge and control of echinococcosis/hydatidosis in hyperendemic areas [38].

## 5. Conclusions

As echinococcosis is still considered one of the 20 neglected tropical diseases of global health importance, it is critical to map its prevalence and create baseline data in order to improve integrated national surveillance. As the true prevalence of echinococcosis is particularly difficult to assess in definitive and intermediate hosts, the need for an integrated surveillance and monitoring system that combines human and animal data is fundamental. This study highlights an underappreciated Public Health issue, allowing the identification of the most problematic areas for echinococcosis in Portugal. This geographic identification allows us to effectively target and carry out educational awareness campaigns, as well as to create management and control guidelines for this zoonosis, namely regular deworming of dogs in close contact with livestock. By comprehensively understanding human cases, hospitalizations, and meat inspection records, the availability of information to decision-makers can be enhanced, leading to the development of improved health policies by both official animal and human health services. This research provides an accurate representation of the situation in Portugal, facilitating the development of effective health control measures, educational programs, and awareness campaigns to address this significant yet neglected zoonotic disease within the framework of One Health.

## Figures and Tables

**Table 1:** Annual data on the number of cases of cystic echinococcosis in bovines, caprines and ovines raised on Portuguese farms, the number of bovines, caprines and ovines slaughtered in Portugal, the officially confirmed cases of human cystic echinococcosis, and the number of human hospitalizations attributable to cystic echinococcosis, in Portugal, from 2008 to 2022.

	Bovine		Caprine		Ovine		Human	
	Positive	Slaughtered	Positive	Slaughtered	Positive	Slaughtered	Cases	Hosp.
2008	3	N/A	0	N/A	0	N/A	N/A	67
2009	2	N/A	0	N/A	0	N/A	4	80
2010	2	405,625	0	114,032	21	995,284	3	77
2011	16	359,614	0	131,916	64	888,660	1	72
2012	21	372,744	0	135,857	22	862,821	2	72
2013	1	332,190	1	120,128	28	866,149	3	46
2014	1	342,220	1	110,685	1	904,196	4	54
2015	3	363,037	1	107,956	1	901,169	5	59
2016	0	377,653	5	102,362	1	839,522	2	39
2017	1	378,466	0	106,900	8	802,263	2	8
2018	4	385,219	0	101,718	2	767,536	9	8
2019	7	374,613	1	100,291	11	791,426	N/A	N/A
2020	12	392,449	1	97,843	18	721661	N/A	N/A
2021	13	418,797	0	104,040	7	768,739	N/A	N/A
2022	9	423,727	1	110,495	8	700,238	N/A	N/A
Total	95	4,926,354	11	1,444,223	192	10,809,664	35	582

N/A—Data not available; Hosp.—Hospitalizations; Total of positive ruminants—298; Total of ruminants slaughtered in Portugal—17,180,241.

## Data Availability

Data are unavailable due to privacy or ethical restrictions.

## Supplementary Materials

The following supporting information can be downloaded at: https://www.mdpi.com/article/10.3390/vetsci10090584/s1, Table S1: Data on the number of cases of cystic echinococcosis in ruminants raised and slaughtered in Portugal, by district, from 2008 to 2022.
