# Supplementary material for: The Evolution of Cystic Echinococcosis in Humans and Ruminants in Portugal—A One Health Approach
Source: Vet Sci. 2023 Sep 21;10(9):584. doi: 10.3390/vetsci10090584 (PMC10537248; doi:10.3390/vetsci10090584)
Supplement: Supplementary file 1 [file vetsci-10-00584-s001.zip › vetsci-2558546-supp.pdf]

Supplementary Table S1. Data on the number of cases of cystic echinococcosis in ruminants raised and slaughtered in Portugal, by district, from 2008 to 2022.

District	Number of Cases
Aveiro	1
Beja	33
Braga	3
SBragança	28
Castelo Branco	6
Coimbra	5
Évora	44
Faro	17
Guarda	12
Leiria	39
Lisboa	1
Portalegre	72
Porto	3
Santarém	22
Setúbal	3
Viana do Castelo	3
Vila Real	2
Viseu	4
**Total**	**298**
